# Adverse Pathological Features and Worse Prognosis in Rectal Cancer Compared With Colon Cancer in T1 and T2 Stages

**DOI:** 10.1002/deo2.70371

**Published:** 2026-07-06

**Authors:** Shunto Iwasaki, Shin‐ei Kudo, Yuta Kouyama, Katsuro Ichimasa, Shigenori Semba, Yuriko Morita, Yuki Takashina, Tomotaka Takizawa, Yu Nimura, Taishi Okumura, Kenta Nakahara, Yasuharu Maeda, Noriyuki Ogata, Takemasa Hayashi, Naruhiko Sawada, Toshiyuki Baba, Tetsuo Nemoto, Masashi Misawa

**Affiliations:** ^1^ Digestive Disease Center Showa Medical University Northern Yokohama Hospital Kanagawa Japan; ^2^ Department of Diagnostic Pathology and Laboratory Medicine Showa Medical University Northern Yokohama Hospital Kanagawa Japan

**Keywords:** rectal cancer, recurrence, T1 colorectal cancer, T2 colorectal cancer, Tis colorectal cancer

## Abstract

**Background and Aim:**

Emerging endoscopic techniques are expanding opportunities for minimally invasive therapy in early colorectal cancers (CRCs). Data regarding the clinicopathological features and outcomes of colon versus rectal cancers in Tis, T1, and T2 stages remain limited. This study evaluates and compares these two entities to clarify their oncologic differences.

**Methods:**

We retrospectively analyzed patients with pTis‐T2 CRCs treated by endoscopic or surgical resection at a single Japanese center between 2001 and 2022. Tumor location was classified as rectum (Ra, Rb) or colon (cecum to RS). Clinical variables (sex and age), pathological features (tumor size, differentiation, lymphatic invasion [Ly], vascular invasion [V], and tumor budding), and recurrence and metastasis rates were compared according to tumor location.

**Results:**

The proportion of rectal cancer rose with invasion depth, from 16.2% (271/1673) in Tis to 18.5% (248/1337) in T1 and 26.4% (178/675) in T2 (*p* < 0.01). Compared with T1 colon cancer, T1 rectal cancer showed higher rates of Ly1‐2 (35.1% vs. 27.4%), V1‐2 (37.9% vs. 24.6%), and recurrence (4.4% vs. 0.6%) (all *p* < 0.05). Similarly, T2 rectal cancer showed higher V1‐2 (68.5% vs. 54.1%) and recurrence rates (13.5% vs. 5.0%) compared with colon cancer (all *p* < 0.05). No significant differences were observed in lymph node or distant metastasis at either T1 or T2 stages.

**Conclusions:**

Rectal cancers demonstrate more aggressive features and higher recurrence rates than colon cancers. Consequently, these patients require more stringent therapeutic approaches and rigorous postoperative surveillance, even in early stages.

**Trial Registration:**

Umin Clinical Trials Registry, UMIN 000042622.

## Graphical abstract

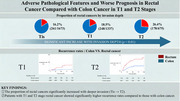

## 1 Introduction

Early‐stage colorectal cancers (CRCs) confined to the mucosa or superficial submucosa can often be removed endoscopically, preserving bowel continuity and avoiding stoma formation [1, 2, 3]. Emerging techniques—such as endoscopic intermuscular dissection, transanal endoscopic myectomy (TEM), and endoscopic full‐thickness resection—are expanding opportunities for minimally invasive therapy in selected T1 and T2 lesions [4, 5, 6, 7]. These organ‐preserving approaches reduce postoperative morbidity and help maintain quality of life. In rectal cancer, advances in endoscopic techniques and chemoradiotherapy have enabled anus‐preserving strategies even for tumors located in the Rb region [6, 7, 8, 9]. Nevertheless, tumor location significantly shapes clinical behavior; rectal cancers exhibit distinct metastatic pathways, higher risks of local recurrence, and unique functional consequences compared with colon cancers, suggesting that omitting surgery remains controversial [2, 10, 11, 12]. As a result, surgical intervention continues to serve as the prevailing therapeutic approach for rectal cancer in current clinical practice.

Recurrence after curative resection and divergent lymphatic spread patterns continue to impede the development of uniform treatment algorithms [13, 14, 15]. Current guidelines are largely based on retrospective series that pool CRCs despite their biological differences. The true oncologic safety margin for local excision of early rectal cancer, therefore, remains uncertain, with reported recurrence rates varying widely. Clearer risk stratification is essential to balance oncologic control with functional preservation.

To address these uncertainties, we conducted a single‐institution retrospective study directly comparing the clinicopathological features and oncologic outcomes of Tis, T1, and T2 CRCs treated with curative intent. The present study aimed to investigate the clinicopathological characteristics and biological aggressiveness of rectal cancer, with a particular focus on the potential applicability of minimally invasive therapeutic approaches.

## 2 Methods

### 2.1 Patients and Study Design

**FIGURE 1:**
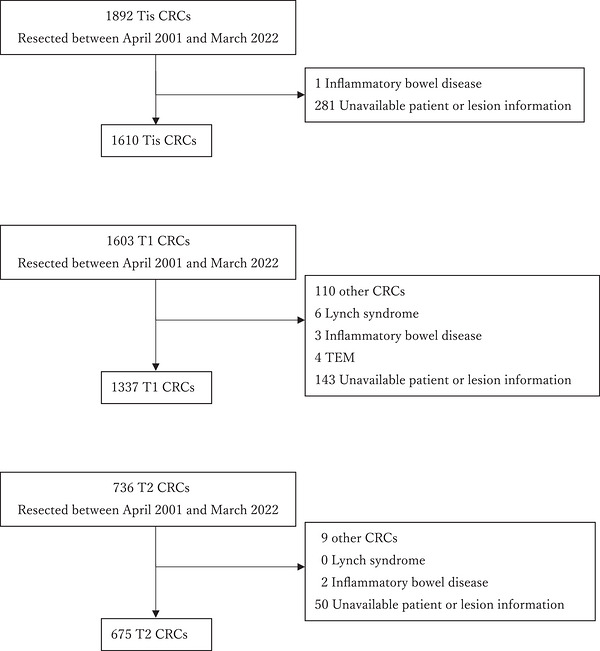
Participant flow diagram. CRC, colorectal cancer; TEM, transanal endoscopic microsurgery. ‘Other CRCs’ denotes cases excluded because of synchronous multiple colorectal cancers other than the lesion analyzed in the present study.

The study cohort comprised patients with pTis, pT1, and pT2 CRCs who underwent treatment at Showa Medical University Northern Yokohama Hospital (Yokohama, Japan) between April 2001 and December 2022. The inclusion criterion was treatment of lesions by endoscopic or surgical resection. The exclusion criteria were inflammatory bowel disease, familial adenomatous polyposis, or Lynch syndrome; treatment by TEM; and concomitant CRCs. TEM was excluded from the research design because it would have compromised the generalizability of the results. Figure 1 shows a patient flow diagram.

The rectum was defined as the region extending from the upper border of the anal canal to the level of the sacral promontory (Ra, Rb). Patient background characteristics analyzed included age, sex, tumor size, histological type, tumor budding, lymphatic invasion (Ly), vascular invasion (V), lymph node metastasis (LNM), and synchronous distant metastasis.

### 2.2 Histological Examination

Resected tissues were fixed in 10% neutral buffered formalin for at least 24 h at room temperature and serially sectioned at 2–3 mm intervals. Specimens were stained with hematoxylin and eosin. Multiple gastrointestinal pathologists made pathological diagnoses. Clinical data were retrospectively collected from medical records. Resection margin status and other relevant histopathological parameters were systematically evaluated in accordance with the Japanese Society for Cancer of the Colon and Rectum (JSCCR) guidelines [16]. Tumor size was defined as the greatest diameter recorded in the original pathology report.

Histological grade was classified following World Health Organization criteria [17]. Grading integrated both the predominant type and the lowest differentiation component. We conducted separate evaluations based on whether the cancer was undifferentiated or not.

V and Ly were assessed by hematoxylin and eosin staining supplemented with Victoria blue staining for V and D2‐40 immunohistochemistry for Ly. V was defined by the presence of neoplastic cells within endothelial‐lined spaces consistent with vascular channels, while Ly was identified as tumor cell infiltration into D2‐40‐positive lymphatic vessels. Based on JSCCR, cases staged as Ly1 and V1 or V2 are treated as Ly+ and V+, respectively [18].

Tumor budding was defined as isolated single cells or clusters of up to five tumor cells at the invasive front. The area with the highest budding density was selected, and buds were counted under a ×20 objective lens. Budding was graded as BD1 (0–4 buds), BD2 (5–9 buds), or BD3 (≥10 buds) in accordance with JSCCR guidelines; BD2 and BD3 were considered budding‐positive [16, 18, 19].

The presence of LNM was confirmed using surgically resected specimens, which served as the reference standard. Accordingly, the association between histological risk factors and LNM was analyzed within the surgical resection cohort.

### 2.3 Treatment Strategy

Most CRCs were evaluated in real time using magnifying chromoendoscopy with 0.40% indigo carmine to determine the pit pattern classification [20, 21]. In addition, we used the JNET classification to support pit pattern diagnosis [20, 22]. Lesions showing a type V pit pattern were further assessed with 0.05% crystal violet. Lesions classified as type III, IV, or V_I_ low‐grade pit patterns were treated by endoscopic resection. Patients with lesions exhibiting a type V_I_ high‐grade or V_N_ pit pattern were referred for surgery [23]. If patients declined surgery, endoscopic resection was used as the first‐line treatment.

According to the Paris classification and JSCCR, T1 CRCs are considered high risk for LNM if it shows undifferentiated type, lymphovascular invasion, a budding grade of ≥2, and a depth of submucosal invasion of >1000 µm [24]. Based on these criteria, we recommend additional surgical resection for T1 cancers with any of these risk factors.

### 2.4 Surveillance and Definition of Recurrence

Postoperative surveillance after surgical resection included measurement of tumor markers every 3 months and computed tomography (CT) every 6 months during the first 3 years, along with the annual colonoscopy. From years 3 to 5, tumor markers were measured every 6 months with annual colonoscopy and CT. Subsequent follow‐up evaluations were continued based on patient preference.

For patients who underwent endoscopic resection, an annual colonoscopy was performed for intramucosal cancers. In patients with T1 cancers treated endoscopically, the follow‐up protocol also included CT and tumor marker measurements, given the potential risk of LNM and distant metastases. If patients declined recommended additional surgery, imaging studies and tumor marker assessments were performed at shorter intervals than standard schedules.

Recurrence was defined as any instance of local recurrence, distant metastasis, or LNM in which recurrence occurred six months later after surgical or endoscopic treatment, and data were collected accordingly. Local recurrence is defined as the detection of a recurrent lesion at the same site as the original treatment. Distant metastasis is defined as the presence of recurrent lesions in other organs, the peritoneum, or lymph nodes.

### 2.5 Statistical Analysis

Values are expressed as mean ± standard deviation and median (interquartile range [IQR]). Dichotomous variables were performed using either Fisher's exact test or Pearson's chi‐squared (χ^2^) test, while continuous variables were analyzed with the Mann–Whitney *U* test. The Cochran‐Armitage trend test was used to evaluate the categorical trends. Cumulative recurrence rates were estimated using the Kaplan–Meier method and compared with the log‐rank test. All statistical analyses were performed using JMP statistical software, version 17.2.0 (SAS Institute Inc., Cary, NC, USA). A two‐sided *p*‐value of <0.05 was considered statistically significant.

### 2.6 Ethical Considerations

This study was approved by the institutional review board of Showa Medical University Northern Yokohama Hospital (approval No. 2024‐013‐A) and was registered with the University Hospital Medical Network Clinical Trials Registry (UMIN 000058035).

## 3 Results

### 3.1 Patient Population

The participant flow diagram is shown in Figure 1. A total of 4231 cases were included in the present study. A total of 609 cases were excluded according to the exclusion criteria. Table 1 shows the clinicopathological characteristics of patients in the present study. In total, 1610 patients exhibited Tis CRCs, 1337 patients exhibited T1 CRCs, and 675 patients exhibited T2 CRCs. The proportion of rectal cancer was 16.2% (261/1610) in Tis, 18.5% (248/1337) in T1, and 26.4% (178/675) in T2. It significantly rose with invasion depth (*p* < 0.01).

**TABLE 1:** Clinicopathological characteristics of patients.

Characteristic	*n* = 3622
Age, years ± SD	66.6 ± 11.6
Tumor size, mm ± SD	23.5 ± 15.0
Sex (Male)	2209 (61.0)
Tis	1610 (44.5)
T1	1337 (36.9)
T2	675 (18.6)

Values are *n* (%) unless otherwise indicated.

### 3.2 Clinical Features of Colon and Rectal Tis Cancers

Table 2 presents the clinicopathological characteristics of the Tis groups. In men, rectal Tis cancers were more frequent than colon cancers. None of the other clinical factors differed between patients with colon and rectal Tis cancers.

**TABLE 2:** Clinicopathological characteristics of patients with colon and rectal Tis cancers.

Characteristic	Colon (*n* = 1349)	Rectum (*n* = 261)	*p*‐value
Age, years ± SD	66.8 ± 12.0	65.8 ± 10.5	0.06
Tumor size, mm ± SD	22.1 ± 14.9	23.6 ± 18.2	0.89
Sex (Male)	841 (62.3)	186 (71.3)	<0.01
Least differentiation (Por or Muc)	0 (0.0)	0 (0.0)	N/A
Predominant differentiation (Por or Muc)	0 (0.0)	0 (0.0)	N/A
Resection margin (+)	11 (0.8)	8 (3.1)	<0.01
Horizontal margin (+)	10 (0.7)	8 (3.1)	<0.01
Vertical margin (+)	1 (0.1)	0 (0.0)	1.00
Depressed type	18 (1.3)	0 (0.0)	0.10
Protruded type	717 (53.2)	141 (54.0)	0.84
Flat type	614 (45.5)	120 (46.0)	0.47
Endoscopic resection <first treatment>	1312 (97.3)	252 (96.6)	0.54
Surgical resection <first treatment>	37 (2.7)	9 (3.5)	0.54
Lymph node metastasis (+)	0 (0.0)	0 (0.0)	N/A
Distant metastasis (+)	0 (0.0)	0 (0.0)	N/A
Local recurrence (+)	0 (0.0)	0 (0.0)	N/A
Distant recurrence (+)	0 (0.0)	0 (0.0)	N/A

Values are *n* (%) unless otherwise indicated.

Abbreviations: Muc, mucinous adenocarcinoma; Por, poorly differentiated adenocarcinoma; S.D., standard deviation.

### 3.3 Clinicopathological Features of Colon and Rectal T1 Cancers

Table 3 presents the clinicopathological characteristics of the T1 groups. Compared with patients with colon T1 cancers, those with rectal ones had larger tumors (26.2 ± 22.5 vs. 20.3 ± 11.2 mm, *p* < 0.01), were younger (64.8 ± 11.2 vs. 67.1 ± 11.4 years, *p* < 0.01), and exhibited higher rates of V (37.9% vs. 24.6%, *p* < 0.01) and Ly (35.1% vs. 27.4%, *p* = 0.02). The rates of LNM (10.2% vs. 11.1%, *p* = 0.73) were similar between rectal and colon T1 cancers. The recurrence rate for patients treated solely with endoscopic therapy was 2.04%, compared to 1.00% for those who underwent surgical resection (*p* = 0.13).

**TABLE 3:** Clinicopathological characteristics of patients with colon and rectal T1 cancers.

	Colon (*n* = 1089)	Rectum (*n* = 248)	*p*‐value
Age, years ± SD	67.1 ± 11.4	64.8 ± 11.2	<0.01
Tumor size, mm ± SD	20.3 ± 11.2	26.2 ± 22.5	<0.01
Sex (Male)	677 (62.2)	156 (62.9)	0.88
Least differentiation (Por or Muc)	149 (13.7)	26 (10.5)	0.21
Predominant differentiation (Por or Muc)	74 (6.8)	14 (5.7)	0.57
Vascular invasion (+)	268 (24.6)	94 (37.9)	<0.01
Lymphatic invasion (+)	298 (27.4)	87 (35.1)	0.02
Tumor budding (grade 2 or 3)	187 (17.2)	55 (22.2)	0.07
Resection margin (+)	67 (6.2)	16 (6.5)	0.88
Vertical margin (+)	59 (5.4)	12 (4.8)	0.88
Horizontal margin (+)	13 (1.2)	6 (2.4)	0.14
Depressed type	246 (22.6)	65 (26.2)	0.24
Protruded type	453 (41.6)	88 (35.5)	0.09
Flat type	390 (35.8)	95 (38.3)	0.47
Endoscopic resection <first treatment>	695 (63.8)	148 (59.7)	0.24
Surgical resection <first treatment>	394 (36.2)	100 (40.3)	0.24
Additional surgical after endoscopic resection ^a^	340 (48.9)	62 (41.9)	0.12
Lymph node metastasis (+) ^b^	75 (10.2)	18 (11.1)	0.73
Lymph node metastasis after additional surgical resection (+) ^c^	39 (11.5)	8 (12.9)	0.83
Distant metastasis (+)	3 (0.3)	0 (0)	1.00
Recurrence <local + distant> (+)	7 (0.6)	11 (4.4)	<0.01
Local recurrence (+)	4 (0.4)	5 (2.0)	0.01
Distant recurrence (+)^d^	3 (0.3)	6 (2.4)	<0.01

^a^ The denominator was calculated exclusively based on cases treated with endoscopic resection. (Colon *n* = 695, Rectum *n* = 148).

^b^ The denominator was calculated exclusively based on cases treated with surgical resection. (Colon *n* = 734, Rectum *n* = 162).

^c^ The denominator was calculated exclusively based on cases treated with additional surgical resection. (Colon *n* = 340, Rectum *n* = 62).

^d^ The organs involved in distant metastatic recurrence were as follows. colon (*n* = 3)—lung 3; rectum (*n* = 6)—lung 3, distant lymph nodes 2, liver 1.

Values are *n* (%) Unless Otherwise Indicated.

Abbreviations: Muc, mucinous adenocarcinoma; Por, poorly differentiated adenocarcinoma; S.D., standard deviation.

### 3.4 Clinicopathological Features of Colon and Rectal T2 Cancers

Table 4 presents the clinicopathological characteristics of the T2 groups. Compared with patients with colon T2 cancers, those with rectal T2 cancers had larger tumors (32.0 ± 13.2 vs. 29.7 ± 13.2 mm, *p* = 0.02), were younger (64.9 ± 11.8 vs. 67.1 ± 11.8 years, *p* = 0.02), and exhibited a higher rate of V (68.5% vs. 54.1%, *p* < 0.01). The rates of LNM (23.6% vs. 27.4%, *p* = 0.37) were similar between rectal and colon T2 cancers. None of the other clinicopathological factors differed between the groups.

**TABLE 4:** Clinicopathological characteristics of patients with colon and rectal T2 cancers.

	Colon (*n* = 497)	Rectum (*n* = 178)	*p‐*value
Age, years ± SD	67.1 ± 11.8	64.9 ± 11.8	0.02
Tumor size, mm ± SD	29.7 ± 13.2	32.0 ± 13.2	0.02
Sex (Male)	249 (50.1)	100 (56.2)	0.19
Least differentiation (Por or Muc)	100 (20.1)	33 (18.5)	0.74
Predominant differentiation (Por or Muc)	16 (3.2)	4 (2.3)	0.61
Vascular invasion (+)	269 (54.1)	122 (68.5)	<0.01
Lymphatic invasion (+)	221 (44.5)	82 (46.1)	0.73
Tumor budding (grade 2 or 3)	43 (8.7)	18 (10.1)	0.55
Resection margin (+)	3 (0.6)	1 (0.6)	1.00
Vertical margin (+)	2 (0.4)	1 (0.6)	1.00
Horizontal margin (+)	1 (0.2)	0 (0.0)	1.00
Endoscopic resection <first treatment>	2 (0.40)	5 (2.81)	0.03
Surgical resection <first treatment>	495 (99.6)	173 (97.2)	0.03
Additional surgical resection after endoscopic resection ^a^	2 (100)	1 (20.0)	1.00
Lymph node metastasis (+)	136 (27.4)	42 (23.6)	0.37
Distant metastasis (+)	6 (1.2)	1 (0.6)	0.68
Recurrence <local + distant> (+)	25 (5.0)	24 (13.5)	<0.01
Local recurrence (+)	1 (0.2)	3 (1.7)	0.06
Distant recurrence (+)^b^	24 (4.8)	21 (11.8)	<0.01
Depressed type	72 (14.5)	29 (16.3)	0.54
Protruded type	109 (21.9)	26 (14.6)	0.04
Flat type	31 (6.2)	10 (5.6)	0.86
Ulcerative type	285 (57.3)	113 (63.5)	0.16

^a^ The denominator was calculated exclusively based on cases treated with endoscopic resection. (Colon *n* = 2, Rectum *n* = 5)

^b^ The organs involved in distant metastatic recurrence were as follows. colon (*n* = 24)—lung 7, liver 13, distant lymph nodes 3, peritoneum 1; rectum (*n* = 21)—lung 8, liver 7, distant lymph nodes 5, peritoneum 1.

Values are *n* (%) Unless Otherwise Indicated.

Abbreviations: Muc, mucinous adenocarcinoma; Por, poorly differentiated adenocarcinoma; S.D., standard deviation.

### 3.5 Recurrence Rates in T1 and T2 Cancers

**FIGURE 2:**
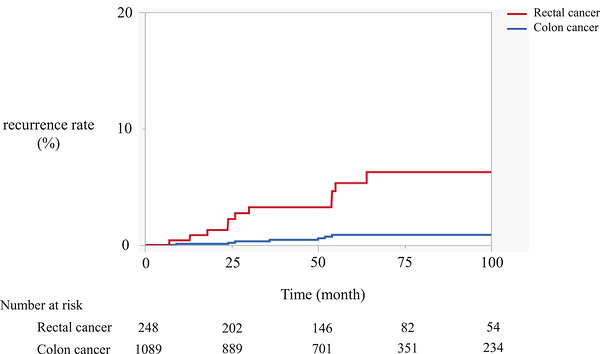
Kaplan‐Meier curves of recurrence rate in pT1 colon and rectal cancers.

**FIGURE 3:**
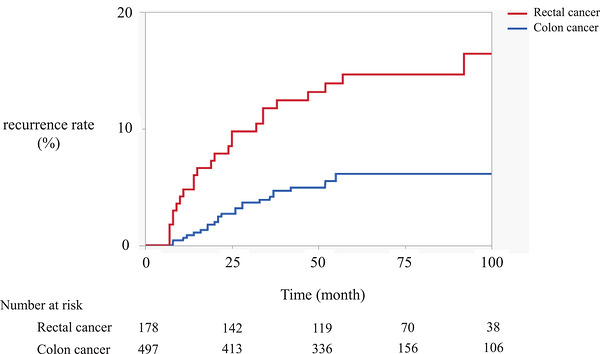
Kaplan‐Meier curves of recurrence rate in pT2 colon and rectal cancers.

Figures 2 and 3 show the Kaplan–Meier curve for recurrence rates in patients with T1 and T2 CRCs. Rectal cancers demonstrated a higher recurrence rate than colon cancers.

Tables 5 and 6 present clinicopathological characteristics of T1 and T2 cancer patients with recurrence and LNM. There was a trend toward a higher local recurrence rate in rectal cancer compared to colon cancer across all T1 cancers, including low‐risk T1 cancers. Furthermore, five out of nine (55.6%) cases were in the rectum among local recurrence cases. Compared to cases without recurrence or LNM, local recurrence cases showed a higher prevalence of rectal localization, undifferentiated‐type carcinoma, and larger tumor size.

**TABLE 5:** Clinicopathological characteristics of T1 cancer patients with recurrence and lymph node metastasis.

	Colon (*n* = 1089)			Rectum (*n* = 248)			Total (*n* = 1337)		
	LNM (+)	Local recurrence (+)	Distant recurrence (+)	LNM (+)	Local recurrence (+)	Distant recurrence (+)	Local recurrence (+)	Distant recurrence (+)	No LNM or recurrence (−)
Characteristic	*n* = 75	*n* = 4	*n* = 3	*n* = 18	*n* = 5	*n* = 6	*n* = 9	*n* = 9	*n* = 1229
Age, years, median [IQR]	63 [54–73]	75 [61–86]	70 [53–82]	59 [54–71]	63 [54–76]	57 [44–74]	68 [60–82]	67 [46–76]	68 [60–75]
Tumor size, mm, median [IQR]	18 [13–23]	28 [18–69]	15 [15–18]	21 [15–28]	23 [20–129]	24 [17–41]	23 [20‐59]	18 [15‐32]	18 [14‐25]
Sex (Male)	37 (49.3)	2 (50.0)	2 (66.7)	9 (50.0)	2 (40.0)	5 (83.3)	4 (44.4)	7 (77.8)	778 (63.3)
Least differentiation (Por or Muc)	20 (26.7)	2 (50.0)	1 (33.3)	1 (5.6)	2 (40.0)	2 (33.3)	4 (44.4)	3 (33.3)	147 (12.0)
Predominant differentiation (Por or Muc)	5 (6.7)	0 (0.0)	0 (0.0)	0 (0.0)	1 (20.0)	2 (33.3)	1 (11.1)	2 (22.2)	80 (6.5)
Vascular invasion (+)	34 (45.3)	0 (0.0)	2 (66.7)	15 (83.3)	2 (40.0)	3 (50.0)	2 (22.2)	5 (55.6)	309 (25.1)
Lymphatic invasion (+)	62 (82.7)	3 (75.0)	3 (100.0)	15 (83.3)	2 (40.0)	5 (83.3)	5 (55.6)	8 (88.9)	297 (24.2)
Tumor budding (grade 2 or 3)	27 (36.0)	1 (25.0)	3 (100.0)	11 (61.1)	1 (20.0)	1 (16.7)	2 (22.2)	4 (44.4)	200 (16.3)
Vertical margin (+)	8 (10.7)	0 (0.0)	0 (0.0)	0 (0.0)	1 (20.0)	0 (0.0)	1 (11.1)	0 (0.0)	62 (5.0)
Horizontal margin (+)	2 (2.7)	1 (25.0)	0 (0.0)	0 (0.0)	1 (20.0)	0 (0.0)	2 (22.2)	0 (0.0)	17 (1.4)
Surgical resection (additional + first)	75 (100.0)	2 (50.0)	2 (66.7)	18 (100.0)	2 (40.0)	3 (50.0)	4 (44.4)	5 (55.6)	797 (64.8)
Endoscopic resection without additional surgery	0 (0.0)	2 (50.0)	1 (33.3)	0 (0.0)	3 (60.0)	3 (50.0)	5 (55.6)	4 (44.4)	432 (35.2)

Values are *n* (%) unless otherwise indicated.

Abbreviations: IQR, interquartile range; LNM, lymph node metastasis; Muc, mucinous adenocarcinoma; Por, poorly differentiated adenocarcinoma.

**TABLE 6:** Clinicopathological characteristics of T2 cancer patients with recurrence and lymph node metastasis.

	Colon (*n* = 497)			Rectum (*n* = 178)			Total (*n* = 675)			
	LNM (+)	Local recurrence (+)	Distant recurrence (+)	LNM (+)	Local recurrence (+)	Distant recurrence (+)	LNM (+)	Local recurrence (+)	Distant recurrence (+)	No LNM or recurrence (−)
Characteristic	*n* = 136	*n* = 1	*n* = 24	*n* = 42	*n* = 3	*n* = 21	*n* = 178	*n* = 4	*n* = 45	*n* = 471
Age, years, median [IQR]	68 [57–76]	75 [75–75]	66 [56–76]	66 [59–74]	62 [59–71]	67 [60–76]	66 [58–75]	66 [60–74]	66 [58–76]	69 [60–75]
Tumor size, mm, median [IQR]	26 [20–33]	30 [30–30]	26 [20–43]	26 [22–35]	26 [24–32]	31 [19–40]	26 [20–33]	28 [24–32]	27 [20–43]	30 [22–36]
Sex (Male)	60 (44.1)	1 (100.0)	15 (62.5)	20 (47.6)	2 (66.7)	8 (38.1)	80 (44.9)	3 (75.0)	23 (51.1)	255 (54.1)
Least differentiation (Por or Muc)	30 (22.1)	0 (0.0)	7 (29.2)	10 (23.8)	1 (33.3)	5 (23.8)	40 (22.5)	1 (25.0)	12 (26.7)	90 (19.1)
Predominant differentiation (Por or Muc)	5 (3.7)	0 (0.0)	2 (8.3)	1 (2.4)	0 (0.0)	0 (0.0)	6 (3.4)	0 (0.0)	2 (4.4)	13 (2.8)
Vascular invasion (+)	96 (70.6)	0 (0.0)	14 (58.3)	35 (83.3)	2 (66.7)	17 (81.0)	131 (73.6)	2 (50.0)	31 (68.9)	244 (51.8)
Lymphatic invasion (+)	128 (94.1)	0 (0.0)	18 (75.0)	38 (90.5)	2 (66.7)	15 (71.4)	166 (93.3)	2 (50.0)	33 (73.3)	125 (26.5)
Tumor budding (grade 2 or 3)	21 (15.4)	0 (0.0)	2 (8.3)	7 (16.7)	1 (33.3)	0 (0.0)	28 (15.7)	1 (25.0)	2 (4.4)	32 (6.8)
Vertical margin (+)	0 (0.0)	0 (0.0)	1 (4.2)	0 (0.0)	0 (0.0)	0 (0.0)	0 (0.0)	0 (0.0)	1 (2.2)	2 (0.4)
Horizontal margin (+)	0 (0.0)	0 (0.0)	0 (0.0)	0 (0.0)	0 (0.0)	0 (0.0)	0 (0.0)	0 (0.0)	0 (0.0)	1 (0.2)

Values are *n* (%) unless otherwise indicated.

Abbreviations: IQR, interquartile range; LNM, lymph node metastasis; Muc, mucinous adenocarcinoma; Por, poorly differentiated adenocarcinoma.

Rectal cancer has a higher rate of distant recurrence compared to colon cancer in both T1 and T2 stages (*p* < 0.01). Among cases involving distant recurrence, lymphatic invasion was positive in 88.9% of T1 cancers and 73.3% of T2 cancers. These proportions appear higher than those in cases without recurrence or LNM. This study found no association between vascular invasion and distant recurrence. When the analysis is restricted to rectal cancers, lymphatic invasion was markedly more frequent in the LNM and distant recurrence groups than in cases without these events, at both T1 and T2 stages. In rectal T1 and T2 cancers, lymphatic invasion was higher in cases with LNM (T1: 83.3% [15/18]; T2: 90.5% [38/42]) or distant recurrence (T1: 83.3% [5/6]; T2: 71.4% [15/21]), compared to cases without these events (T1: 29.9% [66/221]; T2: 30.9% [38/123]).

Concerning distant metastatic recurrence, for T1 tumors, colon cancer recurrences (*n* = 3) occurred solely in the lung, whereas rectal cancers (*n* = 6) involved the lung (*n* = 3), distant lymph nodes (*n* = 2), and liver (*n* = 1). For T2 tumors, colon cancers (*n* = 24) primarily metastasized to the liver (*n* = 13) and lung (*n* = 7), whereas rectal cancers (*n* = 21) most frequently spread to the lung (*n* = 8) and liver (*n* = 7), with both showing minor lymph node and peritoneal involvement.

Compared to the no LNM or recurrence group, the LNM group was characterized by younger age, female predominance, lymphovascular invasion, and Budding Grade≥2.

Among the local recurrence cases of T1 cancer, two cases were positive for horizontal margins, and one case was positive for vertical margins. It was considered that positive surgical margins would be associated with local recurrence. In T2 cancer, no recurrent lesions were observed, except for one case of distant metastasis among those with positive vertical margins.

## 4 Discussion

This study identified several important clinical observations. At the T1–T2 stages, rectal cancers were associated with larger tumor size, a higher incidence of vascular invasion, and a higher recurrence rate than colon cancer. Additionally, the proportion of rectal cancers increases as the depth of invasion progresses from Tis to T1 and T2.

Previous studies showed that rectal cancer has a significantly higher recurrence rate than colon cancer [3, 25]. In patients with Tis who achieved curative resection, no cases of local or distant recurrence were observed. This is consistent with previous reports and further supports the oncological adequacy of endoscopic treatment for intramucosal carcinoma [16]. In both T1 and T2 cancers, rectal localization and lymphatic invasion were identified as potential risk factors for distant metastasis and recurrence. For T1 cancer, tumor size, rectal localization, and histological grade (undifferentiated type) were identified as risk factors for local recurrence. In contrast, no significant risk factors for local recurrence were observed in T2 cancer. High‐risk T1 rectal cancers treated with endoscopic resection alone have also demonstrated higher local recurrence rates than colon cancers [3]. These pathological features may contribute to this difference.

T1 rectal cancers were more likely than T1 colon cancers to exhibit vascular invasion, lymphatic invasion, and a budding grade of ≥2. Previous studies have likewise reported higher rates of vascular invasion in T1 rectal cancers than in T1 colon cancers [2, 3]. Rectal cancers at both T1 and T2 stages also demonstrated larger tumor size, which may be related to the higher prevalence of laterally spreading tumors, granular type (LST‐G), in rectal cancers. Despite these adverse pathological factors, LNM rates did not differ between colon and rectal cancers in either T1 or T2 disease. This may partly reflect the limited number of surgically resected cases included in the analysis. In our cohort, lymphatic invasion was highly positive in colon and rectal cancers with LNM. Previous studies have suggested that lymphatic invasion is the strongest predictor of LNM in T1 cancers, whereas vascular invasion alone is a weak predictor; a similar trend may apply to T2 cancers as well [2, 26]. In our cohort, vascular invasion was more frequent in rectal T2 cancers, yet this did not translate into a higher LNM rate, supporting the idea that vascular invasion may have limited value as a predictive factor for nodal spread in this cohort. Previous studies on T1 Stage found no differences regarding lymphatic invasion [2, 25]. The results suggest that the findings may be influenced by the larger sample size. Regarding T2 cancer, the rate of lymphatic invasion increases in both the colon and rectum as the depth of invasion progresses, which may result in the loss of statistical significance.

The result that the proportion of rectal cancer increased with the depth of invasion may indicate a higher malignant potential of rectal cancer. Previous studies have demonstrated that cancer development in the colon predominantly follows the adenoma–carcinoma sequence, whereas cancer development in the rectum more frequently occurs via the de novo pathway [27, 28]. These differences in underlying mechanisms may partly account for the higher prevalence of advanced‐stage cancer observed in the rectum. Depressed‐type CRCs are presumed de novo in origin [28]. This study shows that depressed cancers were absent in the rectum. Conversely, in T1 and T2 cancers, the rectum showed a higher tendency for depressed‐type lesions compared to the colon. These differences were not statistically significant.

The prevalence of known risk factors, including lymphovascular invasion, histological type, and tumor budding, tended to be higher in the LNM group than in the no recurrence and LNM group [29]. The distinctive dual venous drainage of the rectum—via the portal system (superior rectal vein) and systemic circulation (middle/inferior rectal veins)—facilitates broader hematogenous dissemination [30]. This anatomy likely explains the higher distant recurrence rates in rectal cancers and the tendency, observed in our T2 cases, for colon cancers to metastasize to the liver and rectal cancers to the lung.

This study has several limitations. First, it was a retrospective analysis conducted at a single institution, which may limit the generalizability of the findings. Second, pathological diagnoses and histological features were not re‐evaluated by central review, potentially contributing to interobserver variability. Third, LNM was assessed only in patients who underwent surgical resection, excluding those treated with endoscopic resection alone, so the actual incidence of LNM in the latter group could not be precisely determined. Finally, differences in specimen preparation—specifically, 2‐mm sectioning for endoscopy versus 5‐mm for surgery—may have affected the detection of adverse pathological factors, given the varying proportions of these modalities between colon and rectal groups. These limitations highlight the need for prospective, multicenter studies with standardized pathological review to validate the current treatment strategy.

In conclusion, rectal cancers had higher recurrence rates than colon cancers in T1 and T2 stages. These findings suggest that surgical intervention should be considered in high‐risk cases (undifferentiated type, lymphovascular invasion, budding grade of ≥2) regardless of tumor location and invasion depth. For patients who undergo endoscopic resection alone because of strong personal preference or overall prognosis, comprehensive counseling regarding recurrence risk and postoperative surveillance is essential.

## Author Contributions

*Data curation*: SI, YK, KI, SS, YMo, YT, TT, and YN. *Methodology*: SI, YT, YK, and KI. *Writing – original draft*: SI, YK, and KI. *Resources*: SI, SK, YK, KI, SS, YMo, YT, TT, YN, TO, KN, YMa, NO, TH, NS, TB, TN, and MM. *Investigation*: SI, SK, YK, KI, SS, YMo, and MM. *Supervision*: SK, KI, YMa, NO, TB, and MM. *Project administration*: SK, KI, NS, TB, and MM. *Conceptualization*: YK, KI, and YT. *Writing – review and editing*: YK and KI.

## Funding

This work was supported by JSPS KAKENHI grant number 22K15979.

## Ethics Statement

The Ethics Committee of Showa Medical University Northern Yokohama Hospital 2024‐013‐A.

## Consent

As this was a retrospective study, the requirement for informed consent was waived, and an opt‐out opportunity was provided.

## Conflicts of Interest

The authors declare no conflicts of interest.

## Data Availability

Data are available upon reasonable request.
